# Repetitive Transcranial Magnetic Stimulation Improves Amygdale Functional Connectivity in Major Depressive Disorder

**DOI:** 10.3389/fpsyt.2020.00732

**Published:** 2020-07-31

**Authors:** Fu-jian Chen, Chuan-zheng Gu, Ning Zhai, Hui-feng Duan, Ai-ling Zhai, Xiao Zhang

**Affiliations:** ^1^ Medical Imaging Department，Jining Psychiatric Hospital, Jining, China; ^2^ Psychiatric Department, Jining Psychiatric Hospital, Jining, China; ^3^ Medical Imaging Department, Affiliated Hospital of Jining Medical College, Jining, China; ^4^ Mental Diseases Prevention and Treatment Institute of Chinese PLA, No. 988 Hospital of Joint Logistic Support Force, Jiaozuo, China; ^5^ Mental Rehabilitation Department, Jining Psychiatric Hospital, Jining, China

**Keywords:** major depressive disorder, affective network, repetitive transcranial magnetic stimulation, fMRI, amygdala

## Abstract

Emotional abnormality in major depressive disorder (MDD) is generally regarded to be associated with functional dysregulation in the affective network (AN). The present study examined the changes in characteristics of AN connectivity of MDD patients before and after repetitive transcranial magnetic stimulation (rTMS) treatment over the left dorsolateral prefrontal cortex, and to further assess how these connectivity changes are linked to clinical characteristics of patients. Functional connectivity (FC) in the AN defined by placing seeds in the bilateral amygdale was calculated in 20 patients with MDD before and after rTMS, and in 20 healthy controls (CN). Furthermore, a linear regression model was used to obtain correlations between FC changes and Hamilton depression scale (HAMD) changes in MDD before and after rTMS. Before rTMS, compared with CN, MDD exhibited significantly lower FC between left insula (INS.L), right superior and inferior frontal gyrus (SFG.R and IFG.R), right inferior parietal lobule (IPL.R), and amygdala, and showed an increment of FC between the bilateral precuneus and amygdala in AN. After rTMS, MDD exhibited a significant increase in FC in the INS.L, IFG.R, SFG.R, IPL.R, and a significant reduction in FC in the precuneus. Interestingly, change in FC between INS.L and left amygdala was positively correlated with change in HAMD scores before and after rTMS treatment. rTMS can enhance affective network connectivity in MDD patients, which is linked to emotional improvement. This study further suggests that the insula may be a potential target region of clinical efficacy for MDD to design rationale strategies for therapeutic trials.

## Introduction

Emotional abnormality is a crucial feature of major depressive disorder (MDD) and is also associated with functional dysregulation in the amygdala affective network (AN) (1–3). Furthermore, numerous studies have indicated that brain circuits and network dysfunction can cause depressive symptoms and disability (4, 5). Magnetic resonance imaging (MRI) has been considered to be a non-invasive imaging technology that can help establish relationships between brain circuit and human behavior (5). Therefore, it is essential to understand the relationship between depressive symptom and imaging feature for amelioration in the clinic in the course of MDD.

In resting-state functional connectivity (FC) MRI of MDD, the AN have attracted a great deal of interest. The AN is a frontolimbic circuit comprising brain areas in the amygdala, the subgenual anterior cingulate cortex, the hippocampus, the hypothalamus, orbitofrontal cortex (6, 7), the fusiform gyrus, and the medial frontal gyrus (3). The role of the AN is considered to be the processing emotions and mediating motivated behaviors (6, 7). Recently, numerous MRI studies have reported that MDD patients present dysfunctional AN, including lower FC between the bilateral precuneus and the left amygdala (3), lower FC between the orbitofrontal cortex and the amygdala that are negatively associated with depression scores (8), lower FC between amygdala and posterior insula that are negatively related to increasing severity of behavioral and emotional dysregulation (9), and reduced connectivity between the amygdala and the “positive network” (10). These studies suggest that some crucial brain areas are specifically related to processing and regulating emotions. Indeed, a recently published article has identified symptom-specific targets for MDD, and they thought that different depression symptom had different specific brain circuits (11). However, little is known about the consensus circuit, or the relationship between depressive symptom and AN circuits for amelioration in the clinic.

Transcranial magnetic stimulation (TMS) has been widely used in recent years. Previous studies have widely applied TMS to promote the improvement of cognition, enhancement of neural activity, restoration of network connectivity, and amelioration of negative symptom in neuropsychiatric disorders (4, 12–15), but the mechanism of TMS action is still mostly unclear. Furthermore, some studies have begun to investigate causal relationships between network markers and clinical phenomena in schizophrenia (4). Repetitive transcranial magnetic stimulation (rTMS) is considered as a non-invasive brain stimulation method to modulate cortical excitability (12). Furthermore, rTMS has been approved by the food and drug administration (FDA) to treat the symptoms of MDD. However, it is mostly unknown if AN network target pathology is causally linked to psychiatric symptoms and if the TMS manipulation of network target can reflect the symptom modification of patients with MDD.

The present study examined the changes in the patterns of AN connectivity of patients with MDD before and after rTMS treatment, and further assessed how these connectivity changes are linked to clinical characteristics of patients. We hypothesized that MDD patients would exhibit lower affective network connectivity (for example, lower FC between the amygdala and the insula). We also hypothesized that high-frequency excitatory rTMS could enhance the emotional network connectivity (for example, increase the FC the amygdala and the insula) in MDD patients. Furthermore, it was further hypothesized that changes in AN induced by rTMS are linked to modifications of depressive symptoms of MDD.

## Materials and Methods

### Subjects

Forty MDD patients (who met DSM-5 criteria, right-handed) and 20 healthy controls (CN, right-handed) were recruited from inpatients at the Medical Imaging Department of Jining Psychiatric Hospital (**Table 1**). The inclusion and exclusion criteria of subjects can be seen in the published studies (3, 16, 17). The depressed group consisted of consecutively recruited subjects who met the Diagnostic and Statistical Manual of Mental Disorders, 5th Edition (DSM-5) criteria for major depression without psychotic features and had a score of 20 or higher on the 17-item Chinese Hamilton Depression Scale (HAMD) (18). The healthy controls (CN) were recruited through advertisement and were required to have no history or presence of any psychiatric disorder. The subjects signed written informed consent was taken from all subjects. This study was approved by the Human Participants Ethics Committee at the Medical Imaging Department of Jining Psychiatric Hospital. All patients were randomly assigned to the rTMS group (N = 20) and the sham group (N = 20). The rTMS (or sham) was applied daily on weekdays between Monday and Friday, at the same time of the day, in a course comprising of 25 sessions. Participants received treatment of rTMS or sham over four weeks. Note: the CN subjects were only used to identify brain regions affected by MDD by comparing the network connectivity between CN and MDD and didn’t receive rTMS.

**Table 1:** Demographics and clinical measures of CN, bef-MDD and aft-MDD.

Items	CN	real rTMS MDD		sham rTMS MDD		*F* values (χ^2^)	*p* values
		bef- MDD	aft-MDD	bef- MDD	aft-MDD		
	n = 20	n = 20	n = 20	n = 20	n = 20		
Age (years)	45.95 (8.02)	46.75 (5.52)	46.75 (5.52)	46.30 (4.76)	46.30 (4.76)	0.068	0.991
Sex (male/female)	8/12	9/11	9/11	12/8	12/8	1.735	0.420
Education (years)	11.10 (2.83)	10.40 (1.10)	10.40 (1.10)	10.45 (1.18)	10.45 (1.18)	0.600	0.663
HAMD scores	3.30 (1.53)	26.95 (2.04)^a^	5.75 (2.24)^b,c^	25.50 (2.01)	24.95 (2.24)^d^	666.333	0.000^*^
Duration of illness (months)	NA	25.00 (5.98)	25.00 (5.98)	24.50 (4.76)	24.50 (4.76)	NA	NA
Antidepressant comedication	NA	20(100%)	20 (100%)	20 (100%)	20 (100%)	NA	NA

Data are presented as the mean (with standard deviation, SD). CN, controls; MDD, major depressive disorder; bef-MDD, MDD before treatment; aft-MDD, MDD after treatment; HAMD, Hamilton depression scale. ^*^representing significant differences; ^a^bef-MDD v.s. CN; ^b^aft-MDD v.s. CN; ^c^bef-MDD v.s. aft-MDD; ^d^real rTMS MDD v.s. sham rTMS MDD.

Neuropsychological and MRI assessments were performed on Monday 1 morning at baseline and after 4-week treatment (rTMS or sham). All researchers performing patients’ evaluations were blind to their experimental arm belonging.

Individuals were excluded if they had severe suicide risk, a history of stroke, impaired thyroid function, multiple sclerosis, or degenerative diseases; metastatic cancer, brain tumors, unstable cardiac, hepatic, or renal disease, myocardial infarction, or stroke within the three months preceding the study; metal implants. MDD subjects with history of comorbid Axis I diagnosis were excluded.

### MRI Data Acquisition

MRI images were acquired using a 1.5T Avanto Siemens scanner (Siemens, Erlangen, Germany) with a 12-channel head coil. Resting-state functional images including 240 volumes were obtained by gradient-recalled echo-planar imaging (GRE-EPI) sequence (EPI: TR = 2,000 ms, TE = 25 ms, FA = 90°, matrix = 64 × 64, FOV = 240 mm × 240 mm, thickness = 4.0 mm, no gap, number of slices = 36, voxel size = 3.75 × 3.75 × 4 mm^3^). High-resolution T1-weighted axial images covering the whole brain were acquired by 3D magnetization prepared rapid gradient echo (MPRAGE) sequence (TR = 1,900 ms, TE = 2.48 ms; FA = 9°, matrix = 256 × 256, FOV = 250 × 250 mm, thickness = 1.0 mm, no gap, number of slices = 176, voxel size = 1 × 1 × 1 mm^3^).

### rTMS Protocol

rTMS was delivered using a Magstim Rapid2 magnetic stimulator with a 70-mm figure-8-shaped coil. In all participants, rTMS was applied over the left dorsolateral prefrontal cortex (DLPFC). For stimulation of the DLPFC, the tip of the intersection of the two coil loops was lined up with the F3 sites of the 10–20 electroencephalogram system (19).

The rTMS was applied, using trains of 1,000 stimuli at 10 Hertz (Hz) frequency and an intensity of 90% of the motor threshold (MT). The MT was defined as the lowest intensity (as assessed with single-pulse TMS) producing motor evoked potentials of greater than 50 μV in at least five out of 10 trials in the relaxed first dorsal interosseous (FDI) muscle of the contralateral (right) hand (20). No significant differences were found in MT values between CNs (61 ± 6.3%) and MDD patients (60 ± 7.0%) (CN only perform an MT to compare to MDD and didn’t receive rTMS treatment). Participants received 25 sessions of either rTMS or sham stimulation over the left DLPFC. Each daily stimulation session consisted of 40 trains of 4 s duration with an interval of 56 s. The entire session lasted approximately 25 min twice each day (once at 8 o’clock morning and once at 4 o’clock afternoon), and the interval between daily sessions is 8 h. The total sessions are 1,000 (25 sessions/time * 2 times /day * 5 days/week * 4 weeks), and The total trains are 40000 (40 trains/session * 25 sessions/time * 2 times /day * 5 days/week * 4 weeks). The sham rTMS blocks were conducted, with the coil held close to the DLPFC, but angled away. We chose 90% of MT, although the FDA approved protocol suggests 120% of MT. This reason specified regarding this choice as follows: our subjects were made up of people with 17-item HAMD scores greater than 20, that is, all subjects are major depressive disorder. In the early stages, the use of 120% of MT in some of the patients was attempted, but these subjects felt uncomfortable or emotionally disturbed. Furthermore, some studies have also shown that 90% of MT can improve HAMD scores significantly (21, 22). Finally, to meet safety recommendations, 90% of MT was found most suitable for use.

The sham rTMS group were conducted, with the coil held close to the left DLPFC but angled away (13).

### Image Preprocessing

Data analyses of groups were conducted with Matlab (Math Works Inc., Natick, MA, USA), and Data Processing & Analysis for Brain Imaging (DPABI) (23) based on SPM8. To eliminate T1 equilibration effects, the first ten volumes were discarded. Images were then time-shifted and motion-corrected (24, 25). Participants with head motion more than 2 mm maximum displacement in any direction of x, y, and z or 2◦ of any angular motion throughout the course of scan were excluded from the present study. The resulting images were linearly normalized into Montreal Neurological Institute (MNI) space using a 12-parameter affine approach and an EPI template image. Functional images were resampled to 2 × 2 × 2 mm^3^ voxels and spatially smoothed (6 mm full-width half-maximum Gaussian kernel). Linear detrending and temporal band-pass filtering (0.01–0.08 Hz) were applied to reduce the effect of low-frequency drifts and high-frequency physiological noise. Finally, several nuisance variables, including six head motion parameters, global mean signal (26), CSF signal, and WM signal were removed by multiple linear regression analysis. There were no significant differences between groups in head motion parameters (t = 0.12, p >0.05 for CN vs. MDD; t = 1.16, p >0.05 for pre-rTMS group vs. post- rTMS group; t = 1.34, p >0.05 for pre-sham group vs. post-sham group)) (24, 25).

### Functional Connectivity Analysis

**Figure 1:**
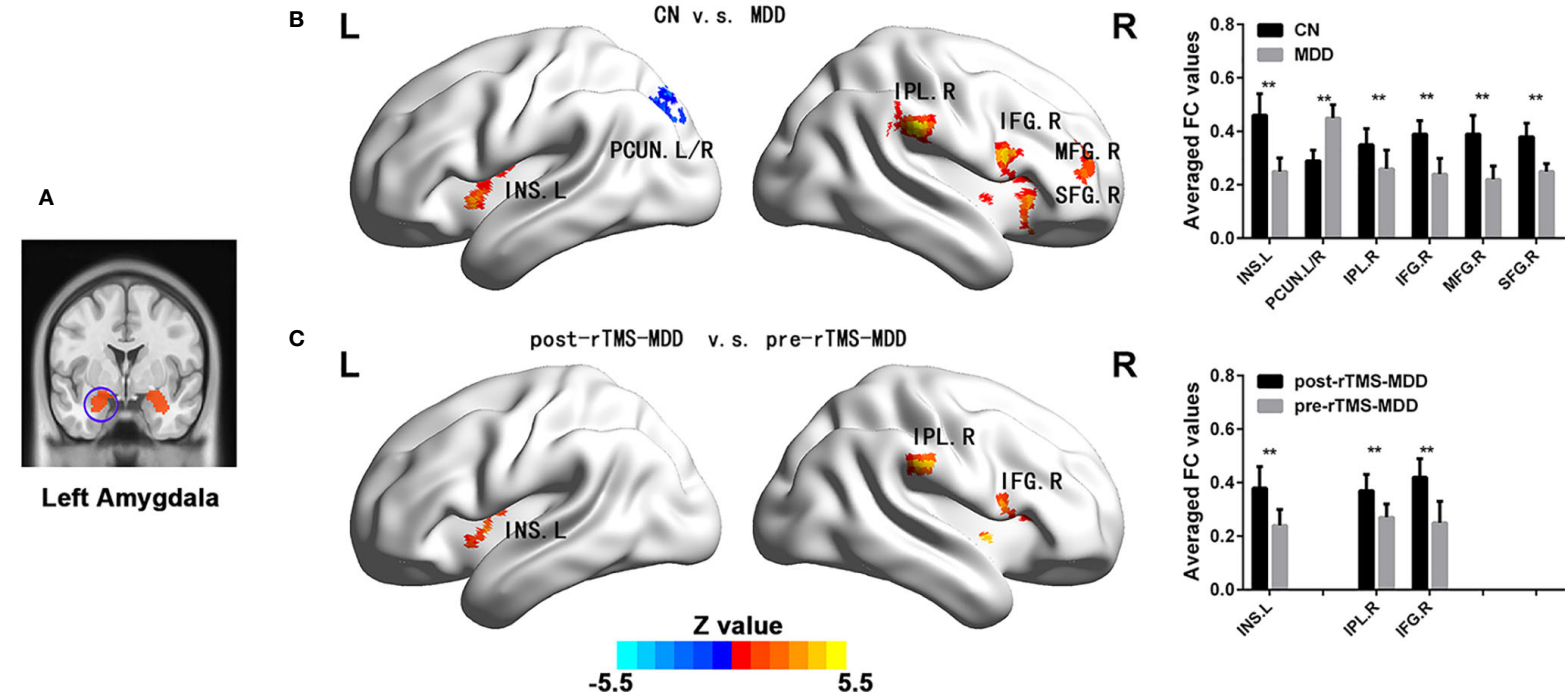
Comparisons of functional connectivity of the left affective network between MDD patients before and after treatment and CN subjects. **(A)** indicating left amygdala ROI. **(B)** brain different regions of the left amygdala functional connectivity between CN and MDD, and pre-rTMS-MDD and post-rTMS-MDD. **(C)** The bar chart shows quantitative differences in left amygdala functional connectivity of these differential brain regions. The statistical maps were managed by False Discovery Rate (FDR) for multiple comparisons to a significant level of p <0.01, with cluster size over 160 mm^3^. CN, controls; MDD, major depressive disorder; pre-rTMS-MDD, MDD before rTMS treatment; post-rTMS-MDD, MDD after rTMS treatment; PCUN.L/R, left/right Precuneus; INS.L, left Insula; IFG.R, right Inferior Frontal Gyrus; IPL.R,right Inferior Parietal Lobule; SFG.R, right Superior Frontal Gyrus. **p <0.01 with FDR for multiple comparisons.

**Figure 2:**
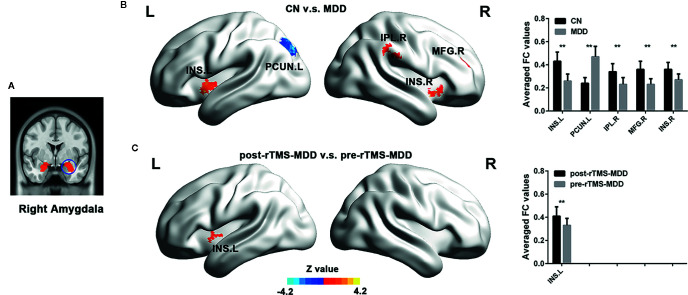
Comparisons of functional connectivity of the right affective network between MDD patients before and after treatment and CN subjects. **(A)** indicating the right amygdala ROI. **(B)** brain different regions of the right amygdala functional connectivity between CN and MDD, and pre-rTMS-MDD and post-rTMS-MDD. **(C)** The bar chart shows quantitative differences in right amygdala functional connectivity of these differential brain regions. The statistical maps were managed by FDR for multiple comparisons to a significant level of p <0.01, with cluster size over 160 mm^3^. CN, controls; MDD, major depressive disorder; pre-rTMS-MDD, MDD before rTMS treatment; post-rTMS-MDD, MDD after rTMS treatment; PCUN.L, left Precuneus; INS.L, left Insula; INS.R, right Insula; IPL.R, right Inferior Parietal Lobul. **p <0.01 with FDR for multiple comparisons.

The seed region of interest (ROI) located in the left and right amygdala (**Figures 1A** and **2A**) was determined by using automated anatomic labelling template (27) implemented with wfu_PickAtlas software and functional connectivity analysis was referred to the previously published study (3). We extracted the individual time courses for each seed as the reference time course, and then performed a voxelwise cross-correlation analysis between the seed region and the whole brain within the grey matter (GM) mask.

### Statistical Analysis

#### Demographic and Neuropsychological Data

The demographic data and clinical variables were compared using two-tailed analysis of variance, Two-samples t-test and chi-square test between MDD and CN.

#### Comparisons of Affective Network Connectivities Between CN and all MDD Subjects

To explain the changes that are related to MDD during rTMS treatment, we performed a voxel-wise analysis. Using a voxel-wise analysis, we compare CN to all 40 MDD (both the rTMS group and the sham group before rTMS) to identify brain regions affected by MDD. Using general mixed linear model analysis, we assessed the differences of the affective network connectivities between all MDD subjects and CN with age, gender, education level and voxel-wise GM volume map treated as covariates. Then a mask was made based on these different brain regions related to MDD. The statistical maps were managed by False Discovery Rate (FDR) for multiple comparisons to a significant level of p <0.01, with cluster size over 160 mm^3^.

#### Comparisons of Affective Network Connectivities Between Pre-rTMS (or Sham) Group and Post- rTMS (or Sham) Group

Using general linear mixed model analysis, we assessed the differences of the affective network connectivities between pre-rTMS group and post- rTMS group (or pre-sham group v.s. post-sham group) within the above-referenced mask with age, gender, education level and voxel-wise GM volume map treated as covariates. The statistical maps were managed by FDR for multiple comparisons to a significant level of p <0.01, with cluster size over 160 mm^3^.

#### Correlations Between Changes of Connectivity and HAMD Scores Pre- Versus Post-rTMS

For each participant, the change in affective network connectivity was calculated to examine the correlations between FC changes post- versus pre-rTMS and HAMD scores pre- versus post-rTMS. The relationships between FC changes and HAMD changes in MDD before and after rTMS was also investigated using a linear regression model (independent variable: FC changes, dependent variable: HAMD changes).

## Results

### Demographic and Neuropsychological Characteristics

No significant differences were observed in age, gender, or education level between the MDD patients and CN groups (all p >0.05, **Table 1**).

### Comparison of Affective Network Connectivities Between CN and All MDD Subjects

In the left affective network, compared with CN, MDD subjects showed significantly lower FC between left insula (INS.L), right inferior frontal gyrus (IFG.R), right superior frontal gyrus (SFG.R), right inferior parietal lobule (IPL.R), right middle frontal gyrus (MFG.R) and left amygdala, and showed significantly higher FC between bilateral PreCUN and left amygdala (**Figures 1A, B** and **Table 2**).

**Table 2:** Functional connectivity of the affective network between MDD and CN, and bef-MDD and aft-MDD.

Brain region	BA area	Peak MNI coordinate			Peak *Z* value	Cluster size (mm^3^)
		x	y	z		
**The left affective network**						
**(1) CN < MDD**						
L Precuneus	19	−24	−78	48	−4.9316	156
R Precuneus	30	15	−45	9	−5.3528	278
** CN > MDD**						
L Insula	13	−36	9	−3	4.3346	159
R Inferior Frontal Gyrus/Insula	13	39	15	12	5.3433	477
R Superior Frontal Gyrus	8	3	30	45	4.2349	171
R Inferior Parietal Lobule	40	57	−27	30	4.7245	193
R Middle Frontal Gyrus	10	42	39	−6	3.8478	64
**(2) bef- MDD < aft- MDD**						
L Insula	13	−36	3	0	4.136	55
R Inferior Frontal Gyrus/Insula	13	48	15	−3	5.0377	146
R Inferior Parietal Lobule	40	60	−24	30	4.7197	55
**The right affective network**						
**(1) CN < MDD**						
L Precuneus	19	−24	−78	51	−4.1022	175
** CN > MDD**						
L Insula	13	−36	6	−9	4.0263	54
R Inferior Frontal Gyrus/Insula	13	36	9	−12	3.346	32
R Inferior Parietal Lobule	40	63	−33	27	3.9365	82
R Middle Frontal Gyrus	10	36	51	21	3.0563	30
**(2) bef -MDD < aft- MDD**						
L Insula	13	−36	3	0	3.4073	30

CN, controls; MDD, major depressive disorder; bef-MDD, MDD before treatment; aft-MDD, MDD after treatment.

In the right affective network, compared with CN, MDD subjects exhibited significantly lower FC between INS.L, IFG.R/INS.R, IPL.R, MFG.R and right amygdala, and exhibited a significantly higher FC between left precuneus (PCUN.L) and right amygdala (**Figure 2B** and **Table 2**).

### Comparison of Affective Network Connectivities Between Pre-rTMS (or Sham) and Post-rTMS (or Sham) in MDD Patients

In the left affective network, compared with pre-rTMS-MDD, post-rTMS-MDD exhibited a significant increase of FC between INS.L, IFG.R/ right insula (INS.R), IPL.R and left amygdala (**Figures 1A, C** and **Table 2**). Compared with post-sham-rTMS-MDD, post-rTMS-MDD exhibited a significant rise in FC between INS.L, IFG.R/ right insula (INS.R), IPL.R and left amygdale.

In the right affective network, compared with pre-rTMS-MDD, post-rTMS-MDD exhibited a significant increase of FC between INS.L and right amygdala (**Figures 2A, C** and **Table 2**). Compared with post-sham-rTMS-MDD, post-rTMS-MDD exhibited a significant increase of FC between INS.L and right amygdala.

Furthermore, there were no changes for AN functional connectivity between pre-sham group vs. post-sham group.

### Correlations Between Changes of Connectivity and HAMD Scores Pre- Versus Post-rTMS or Pre-Sham Versus Post-Sham-rTMS

**Figure 3:**
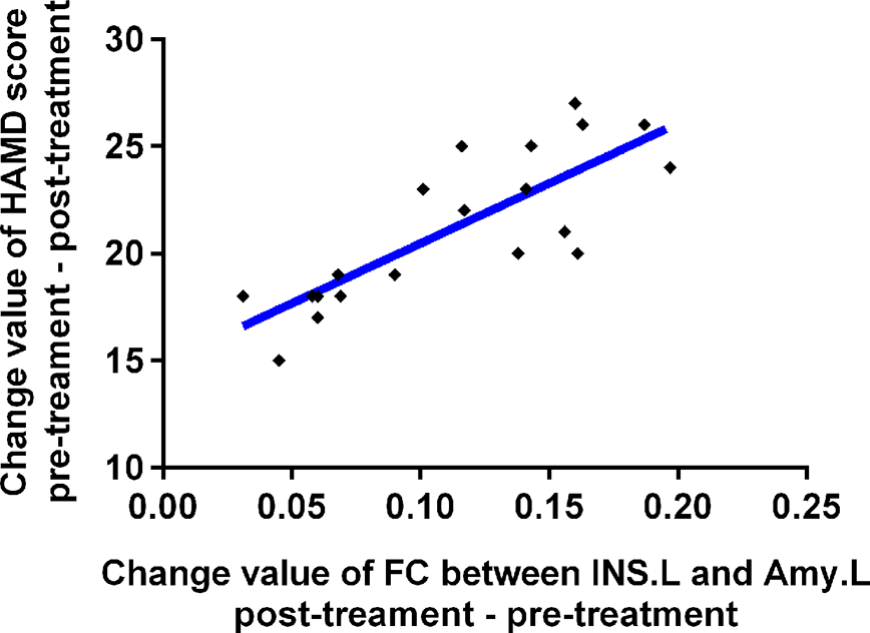
Relationships between change value of HAMD scores and change value of affective network connectivity between MDD patients before and after treatment. MDD, major depressive disorder; FC, functional connectivity; bef-MDD, MDD before treatment; aft-MDD, MDD after treatment; INS.L, left Insula; Amy.L, left Amygdala; HAMD, Hamilton depression scale.

As shown in **Figure 3**, the change in FC between INS.L and left amygdala was positively correlated with the changes in HAMD scores before and after TMS treatment (p <0.001). Furthermore, there were no correlations between changed FC and the changes in HAMD scores (25.50 v.s. 24.95) before and after sham TMS treatment (p >0.05).

## Discussion

This study investigated the changing patterns of amygdala connectivity of MDD patients before and after rTMS treatment, and illuminated brain circuit or the relationship between the network and human behaviour in MDD. Firstly, compared to CN, MDD subjects showed significantly lower FC between INS.L, IPL.R, IFG.R, MFG.R, SFG.R and amygdala, and exhibited significantly higher FC between the bilateral precuneus and the left amygdala before rTMS. Secondly, after rTMS, MDD showed a significant increased FC in the INS.L, IFG.R/INS.R, and IPL.R. Finally, changes in FC between INS.L and left amygdala were positively correlated with changes in HAMD scores before and after rTMS treatment. Altogether, this study promotes our further understanding of the possible anatomical basis of MDD associated with emotional dysregulation.

Before rTMS treatment, this study exhibited lower FC between the left insula and amygdala in MDD patients, which are in substantial agreement with previous studies (9). It has been indicated that emotional dysregulation in MDD patients is related to FC between amygdala and insula supporting emotion regulation (28). Some anatomical studies have reported that anterior insula is connected with the amygdala by subgenual ACC, which plays a crucial role in generating negative mood states (29). Functional neuroimaging studies in the resting brain have also demonstrated that the anterior insular cortex subserves the emotional functions (30, 31). Furthermore, the present study also exhibited lower FC between the right superior and inferior frontal gyrus, right inferior parietal lobule and amygdala. Our findings also suggest that MDD patients show an impairment of salience and emotion perception, and impairment of emotional controlling and goal-directed behavior, which is in concordance with previous studies (30, 32, 33). Indeed, some recent studies have also reported the therapeutic effects in AN (34) and frontostriatal network (35) in patients with MDD. However, our findings are inconsistent with a recent study (34), who investigated the effect of rTMS on neural connections in depressed patients. Although they also showed changes in amygdala connectivity, their findings showed the more subgenual anterior cingulate cortex role while our study mainly found reduced connectivities of the left insula, right superior and inferior frontal gyrus, right inferior parietal lobule in AN. This reason for the differences may be as follows. The disease severity of our subjects (mean HAMD-17 score: 26.95 for active rTMS, and 25.5 for sham rTMS) were more severe than those of their study (mean HRSD-17 score: 16 for active rTMS, and 13.1 for sham rTMS). Indeed, a series of studies have shown different emotional processing between minor depression and major depression and different mechanisms of electrophysiological damage (36).

Interestingly, our study showed increased FC between the bilateral precuneus and amygdala in MDD patients, which indicates increased connectivity could be associated with the persistently negative mood state and rumination (3). The precuneus is consistently considered to participate in the processing of episodic memory (37) and to be implicated in the encoding of self-relevant information (38, 39). A large number of studies have indicated that the precuneus is a crucial node of the so-called default mode network (DMN) (40, 41). Accumulating evidence has indicated that the DMN is involved in self-referential processing, including self-prospecting and internal monitoring, autobiographical memory retrieval, future planning, and theory of mind (40, 42, 43). Therefore, our findings further suggest that patients with MDD may fail to restrain negative self-thoughts (44). This finding is inconsistent with our earlier results done at another research centre, which showed reduced FC between the left amygdala and the bilateral precuneus in MDD patients (3). It could be due to the difference in the mean age of subjects in the current study (46.75 years) v.s. the previous studies (32.11 years). Indeed, previous studies have reported that MDD presented age-related abnormalities of grey and white matter (45), and age-related changes in physiological functioning (46). Furthermore, the mean duration of illness in this study (25.00 months) was longer than those of previous studies (mean = 0.7 years). Previous studies have also indicated that duration of illness can affect adverse consequences in terms of outcome for individuals with MDD (47). Therefore, it is reasonable to assume that these inconsistencies in results are due to differences in age of subjects and the duration of their illness.

After rTMS treatment, our study showed significantly increased FC in these above-mentioned brain regions (INS.L, IFG.R, SFG.R, and IPL.R) showing altered connectivity in the affective network. Furthermore, these frontoparietal structures are classified as domain-general control regions, that is so-called cognitive control network (48, 49). Our findings suggest that rTMS can improve functional connectivities of brain regions in interaction hubs between the affective network and executive function network in MDD patients, which provides a potentially reproducible model of TMS-based rescue of a breakdown of connectivity. Some studies have reported that insula is connected with prefrontal cortices, which plays a role in emotional controlling and goal-directed behaviour (33). AN is considered to participate in emotional processing and mediating motivated behaviors (7, 50), emotional controlling and goal-directed behaviour (33). Meanwhile, orbital fronto-insular cortices are part of both the salience network and the AN (51). A recently published article has identified symptom-specific targets for MDD, and they thought that different depression symptom had different specific brain circuits (11). It is reasonable to speculate that the AN might be partially connected with the salience network in MDD patients. The excitatory rTMS plays a therapeutic improvement role in through stimulation of the DLPFC, which is connected with the salience network and the AN. Altogether, our findings suggest that non-invasive treatment of AN network dysfunction by stimulating the DLPFC is an effective strategy to improve depressive symptoms in MDD patients.

Interestingly, this study also found that changes in FC between INS.L and left amygdala was positively correlated with changes in HAMD scores before and after rTMS treatment, which indicates the existence of at least one network circuit linked directly to depressive symptoms. Indeed, it has been hypothesized that emotional dysregulation is considered to contribute to altered FC between the amygdala and insula (28), and it is reported that an insula-centered neural network is considered to support salience and emotion perception (30, 32). The insula is also believed to be connected with prefrontal cortices, which plays a role in emotional controling and goal-directed behavior (33). Therefore, it is reasonable to speculate that the rTMS-induced improvement of depressive symptoms supports the standpoint that DLPFC is directly implicated in the onset of depressive symptom in MDD patients. According to a neurobiological view, rTMS may induce relevant modulations to activate the changes in synaptic plasticity over a DLPFC–amygdala–insula circuit, which leads to clinical improvement. Therefore, the precise localiZation of a DLPFC–amygdala–insula circuit is critical in identifying the underlying neural substrates of the disabling depressive symptoms in patients with MDD. Our results provide experimental evidence in support of such a circuit, which, when directly modulated, rescues these deficits.

However, this has some limitations. Firstly, the longitudinal data was not available for the controls. In future studies, the longitudinal data of controls should be designed to ensure that the network changes observed pre/post-treatment are due to rTMS and not merely within the range of normal variation in FC, although FC is not expected to change within a short period. Secondly, this sham stimulation may affect our findings. The current sham rTMS studies are conducted using coils specially designed to deliver sham stimulation. However, tilting the coil away from DLPFC still provides low-intensity stimulation due to the magnetic field at the edge of the coil. Therefore, this limitation should be kept in mind. Finally, amygdala network connectivity is not the same as AN connectivity. The amygdala is associated with multiple functional networks, including the so-called salience network, which is involved in attentional and cognitive control in domains beyond affect. Although we have explained that TMS can improve functional connectivities of brain regions in interaction hubs between amygdala affective network and other networks (i.e. executive function network, DMN, and salience network and so on) in MDD patients, our interpretation of the results needs to be cautious.

The present study provides novel evidence that rTMS may be an effective strategy for improving the depressive symptom of patients with MDD, which may be useful in further characterizing network–symptom relationship in MDD. While our results demonstrate a target biological substrate for the treatment of depressive symptoms, we do not suggest that DLPFC–amygdala–insula-targeted TMS is the sole intervention that can do so. Our results raise the possibility that functional connectivity may be a useful marker of efficacy for other therapeutic interventions in the treatment of depressive symptoms. It further suggests that the amygdala–insula circuit may be a potential interventional target circuit of clinical efficacy for MDD to design rationale strategies for therapeutic trials.

## Data Availability Statement

The datasets generated for this study are available on request to the corresponding authors.

## Ethics Statement

The studies involving human participants were reviewed and approved by Human Participants Ethics Committee at the Medical Imaging Department of Jining Psychiatric Hospital. The patients/participants provided their written informed consent to participate in this study.

## Author Contributions

F-JC, C-ZG, H-FD, and XZ contributed study concept and design. NZ, H-FD, A-LZ, and XZ acquired, analyzed, or interpreted data. F-JC and H-FD drafted and revised the manuscript. All authors contributed to the article and approved the submitted version.

## Conflict of Interest

The authors declare that the research was conducted in the absence of any commercial or financial relationships that could be construed as a potential conflict of interest.
